# Rhodamine B Photodegradation in Aqueous Solutions Containing Nitrogen Doped TiO_2_ and Carbon Nanotubes Composites

**DOI:** 10.3390/molecules26237237

**Published:** 2021-11-29

**Authors:** Adelina Udrescu, Stefania Florica, Madalina Chivu, Ionel Mercioniu, Elena Matei, Mihaela Baibarac

**Affiliations:** 1 Laboratory of Optical Processes in Nanostructured Materials, National Institute of Materials Physics, Atomistilor Street 405A, POB MG 7, 077125 Bucharest, Romania; stefania.florica@infim.ro (S.F.); madalina.chivu@infim.ro (M.C.); 2 Laboratory of Atomic Structures and Defects in Advanced Materials, National Institute of Materials Physics, Atomistilor Street 405A, POB MG 7, 077125 Bucharest, Romania; imercioniu@infim.ro; 3 Laboratory of Multifunctional Materials & Structures, National Institute of Materials Physics, Atomistilor Street 405A, POB MG 7, 077125 Bucharest, Romania; elena.matei@infim.ro

**Keywords:** titanium dioxide, carbon nanotubes, UV–VIS spectroscopy, photocatalytic properties

## Abstract

In this work, new results concerning the potential of mixtures based on nitrogen doped titanium dioxide (TiO_2_:N) and carbon nanotubes (CNTs) as possible catalyst candidates for the rhodamine B (RhB) UV photodegradation are reported. The RhB photodegradation was evaluated by UV–VIS absorption spectroscopy using samples of TiO_2_:N and CNTs of the type of single-walled carbon nanotubes (SWNTs), double-wall carbon nanotubes (DWNTs), multi-wall carbon nanotubes (MWNTs), and single-walled carbon nanotubes functionalized with carboxyl groups (SWNT-COOH) having various concentrations of CNTs. The best photocatalytic performance was obtained for sample containing TiO_2_:N and 2.5 wt.% SWNTs-COOH, when approx. 85% of dye removal was achieved after 300 min. of UV irradiation. The reaction kinetics of RhB aqueous solutions containing TiO_2_:N/CNT mixtures followed a complex first-order kinetic model. The TiO_2_:N/CNTs catalyst induced higher photodegradation efficiency of RhB than TiO_2_:N due to the presence of CNTs, which act as adsorbent and dispersing agent and capture the photogenerated electrons of TiO_2_:N hindering the electron–hole recombination.

## 1. Introduction

Titanium dioxide (TiO_2_) is a wide bandgap semiconducting material (3.0, 3.2, and 3.5 eV for rutile, anatase, and brookite phases, respectively) that has gained special attention due to its nontoxicity, and chemical and physical stability [1,2]. TiO_2_ is the most used n-type semiconductor in different environmental applications and energy fields, such as photocatalysis and dye-sensitized solar cells [3]. Great efforts have been made to solve the water pollution issues; therefore, the main objective is focused on the removal of dyes from wastewater [4]. In order to remove the pollutants, the methods used involve biodegradation, adsorption, and membrane filtering [5]. A practical method for the removal of pollutants considers the use of sunlight for photodegradation of dyes, and the main process involves advanced oxidation. The principal mechanism of advanced oxidation implies the generation of hydroxyl radicals, which are responsible for the degradation of organic pollutants. The ability of materials to be photoactivated by sunlight implies a good knowledge of the charge transport processes that take place. Thus, the charge transfer induced by UV irradiation has been studied using various experimental techniques such as photoluminescence, theoretical modeling, etc.

The photocatalytic activity of TiO_2_ is limited by the band edge absorption at the UV region and by the fast electron–hole recombination, which leads to the decrease of quantum efficiency. To improve the photocatalytic activity of TiO_2_, it is recommended to modify its structure to operate in natural sunlight conditions. TiO_2_ can be modified using various techniques: doping with metals or non-metals (nitrogen [6], sulfur [7], fluorine [8], etc.) or combining with carbonaceous materials [9,10,11,12,13]. Doping and co-doping of TiO_2_ induces the narrowing of the bandgap (from 3.2 eV to 1.51 eV [14]) and lowering the electron–hole recombination rate [14]. From doping with various non-metal elements, nitrogen doping is the most effective due to its chemical, structural, and electrical properties [15]. Recently, a narrowing of the bandgap has been reported when the nitrogen doped TiO_2_ (TiO_2_:N) was mixed with SiO_2_ nanoparticles [16]. The resulting blend was reported to have self-cleaning properties when this was deposited onto cotton fabrics colored with methylene blue and forest fruits juice, respectively, in the presence of UV light [16]. Carbon nanotubes have good tensile strength and Young’s modulus, and due to their larger specific surface area, they are often used in combination with TiO_2_ [17]. CNTs play the role of charge acceptor; therefore, they can accept photo-excited electrons, leading to the delay of exciton recombination [13].

Only a few studies performed on the photocatalytic degradation of rhodamine B (RhB) using as catalyst TiO_2_:N have been reported thus far [18,19,20]. A sustained effort was carried out for synthesis of the composites based on TiO_2_:N and carbon nanotubes of the type single-walled carbon nanotubes (SWNTs), multi-wall carbon nanotubes (MWNTs), and MWNTs functionalized with carboxyl, carbonyl, and hydroxyl groups [10,11,12,13,17,21,22,23,24], as well as their applications in the degradation of various pollutants such as phenol [24] or dyes, including methylene blue [17], methylene orange [17], orange G [21], Congo red [21], and Indigo Carmin [21]. In contrast with this progress, the main novelty elements which will be reported in this work regard the following: (i) RhB photodegradation in the presence of TiO_2_:N/SWNT, TiO_2_:N/DWNT, and TiO_2_:N/MWNT composites; and (ii) the influence of the carboxyl groups onto SWNT surfaces dispersed into the TiO_2_:N matrix. In this context, this study is focused on the following important aspects regarding the photodegradation of RhB in aqueous solutions containing TiO_2_:N/CNT composites: (i) the determination of the optimal single-walled carbon nanotube (SWNT) concentration in TiO_2_:N/SWNT composites to obtain the best photocatalytic activity; (ii) the influence of CNTs, including double-wall carbon nanotubes (DWNTs), multi-wall carbon nanotubes (MWNTs), and single-walled carbon nanotubes functionalized with carboxyl groups (SWNTs-COOH), on the photocatalytic efficiency of composites based on TiO_2_:N/CNTs; and (iii) the explanation of the rate kinetic mechanism of RhB aqueous solutions in the presence of TiO_2_:N/SWNTs-COOH mixtures.

## 2. Results

### 2.1. Optical Properties of the TiO_2_:N/CNTs Samples

Seven samples were used for the RhB photodegradation, characterized by the compositions shown in Table 1.

**Figure 1:**
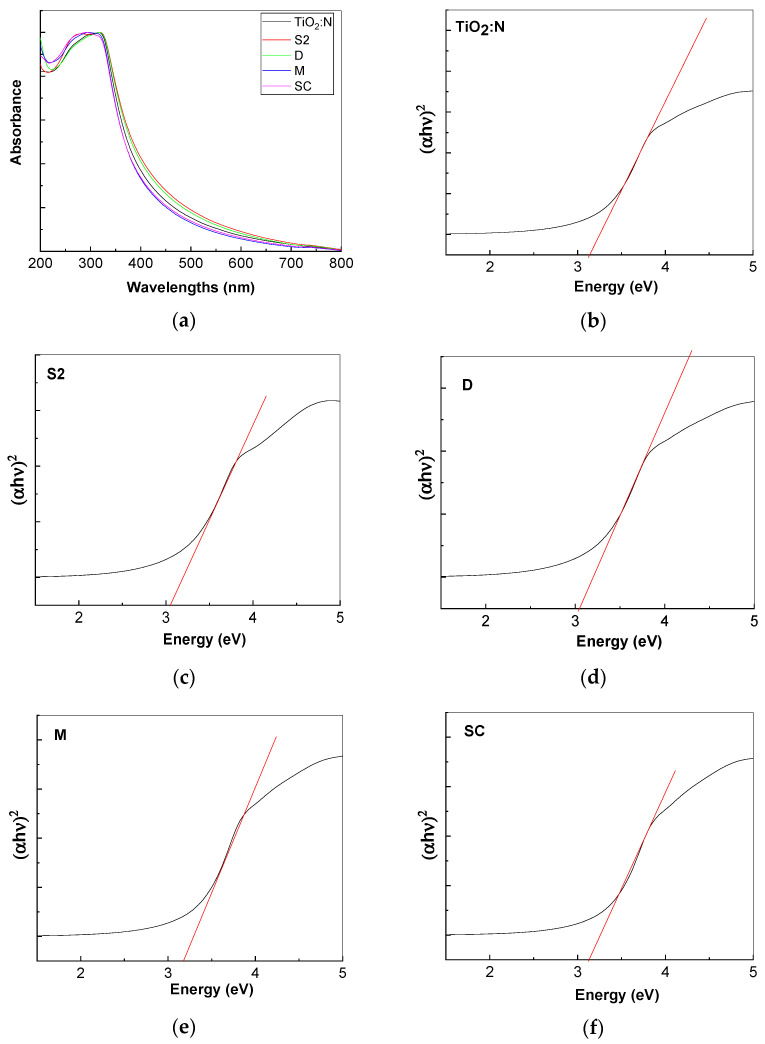
UV–VIS spectra (**a**) and the Tauc’s plots for the assessing of the band gap of the samples of TiO_2_:N (**b**), TiO_2_:N/SWNTs (**c**), TiO_2_:N/DWNTs (**d**), TiO_2_:N/MWNTs (**e**), and TiO_2_:N/SWNTs-COOH (**f**). The abbreviations M, SC, D and S2 correspond to samples with the composition shown in Table 1.

Figure 1 shows the UV–VIS spectra (a) and the Tauc’s plots for the assessing of the band gap (E_g_) of the samples of TiO_2_:N (b), TiO_2_:N/SWNTs (c), TiO_2_:N/DWNTs (d), TiO_2_:N/MWNTs (e), and TiO_2_:N/SWNTs-COOH (f). The values of E_g_ in the case of the samples TiO_2_:N, S2, D, M, and SC were equal to 3.17 eV, 3.04 eV, 3.06 eV, 3.15 eV, and 3.12 eV, respectively. The presence of carbon nanotubes induced a small narrowing of the band gap of the TiO_2_:N nanoparticles. The large E_g_ of TiO_2_:N nanoparticles allowed an efficient absorption only in the UV light region.

**Figure 2:**
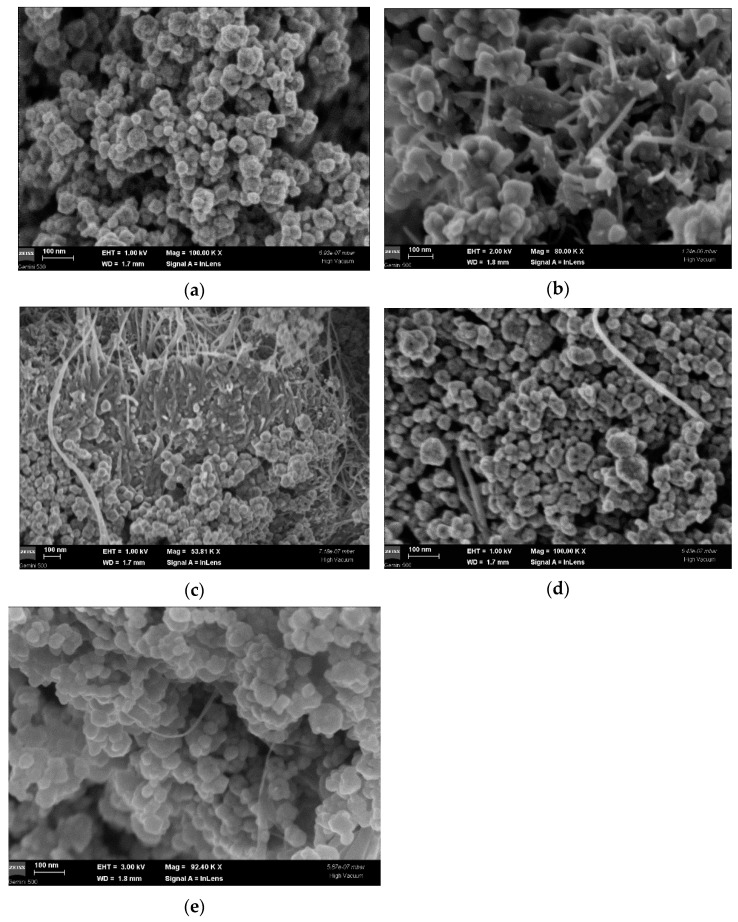
SEM images of the samples of TiO_2_:N (**a**); TiO_2_:N/SWNTs (S2 sample) (**b**); TiO_2_:N/DWNTs (D sample) (**c**); TiO_2_:N/MWNTs (M sample) (**d**); and TiO_2_:N/SWNTs-COOH (SC sample) (**e**).

**Figure 3:**
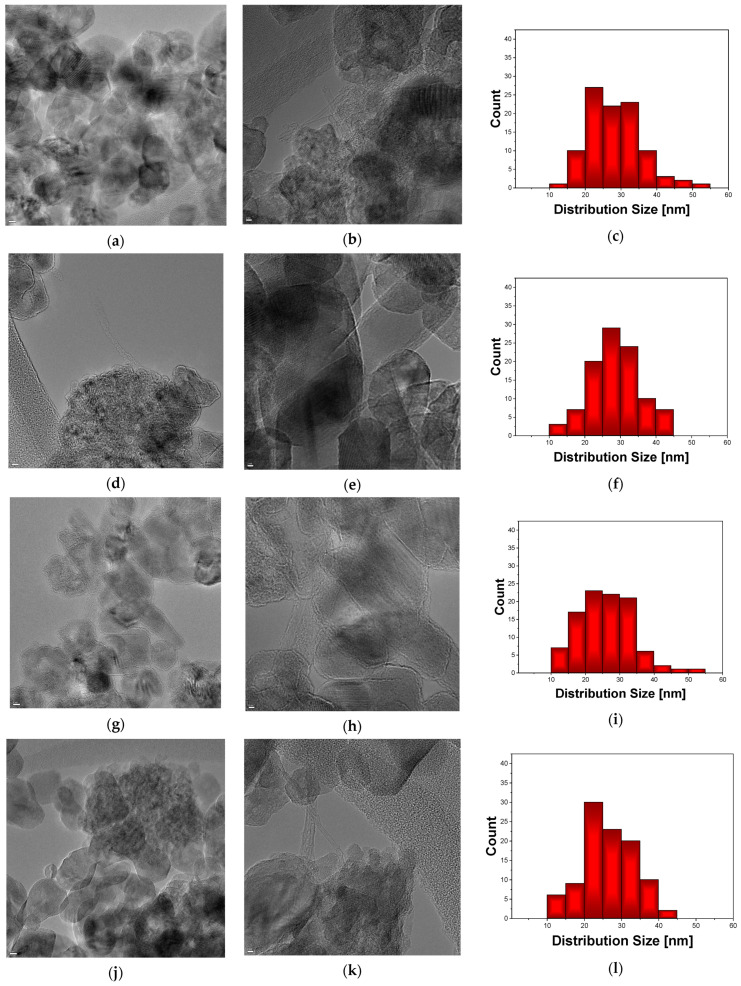
TEM images with the magnification 500 kX and 1M as well as the TiO_2_:N size distribution in the case of the samples of TiO_2_:N/SWNTs (S2 sample; **a**–**c**) TiO_2_:N/DWNTs (D sample; **d**–**f**), TiO_2_:N/MWNTs (M sample; **g**–**i**) and TiO_2_:N/SWNTs-COOH (SC sample; **j**–**l**).

The SEM analysis highlights in Figure 2 the TiO_2_:N irregular platelets, while in the case of the S2, D, M, and SC samples, both TiO_2_:N platelets and one-dimensional structures corresponding to the bundles of SWNTs, DWNTs, MWNTs, and SWNTs-COOH were observed. Figure 3 shows the TEM images of the S2, D, M, and SC samples as well as the size distribution of TiO_2_:N nanoparticles in the S2, D, M, and SC samples. For statistical purposes, 100 particles were measured for each sample. Mean sizes of TiO_2_:N nanoparticles in the S2, D, M, and SC samples were equal to 28.4 nm, 28.81 nm, 26.11 nm, and 26.43 nm, respectively. The minimum and maximum sizes, respectively, of the TiO_2_:N nanoparticles in the case of the samples labeled were as follows: (i) S2, 10.87 nm and 62.39 nm; (ii) D, 13.57 nm and 44.81 nm; (iii) M, 10.06 nm and 52.92 nm; and (iv) SC, 12.74 nm and 42.24 nm.

**Figure 4:**
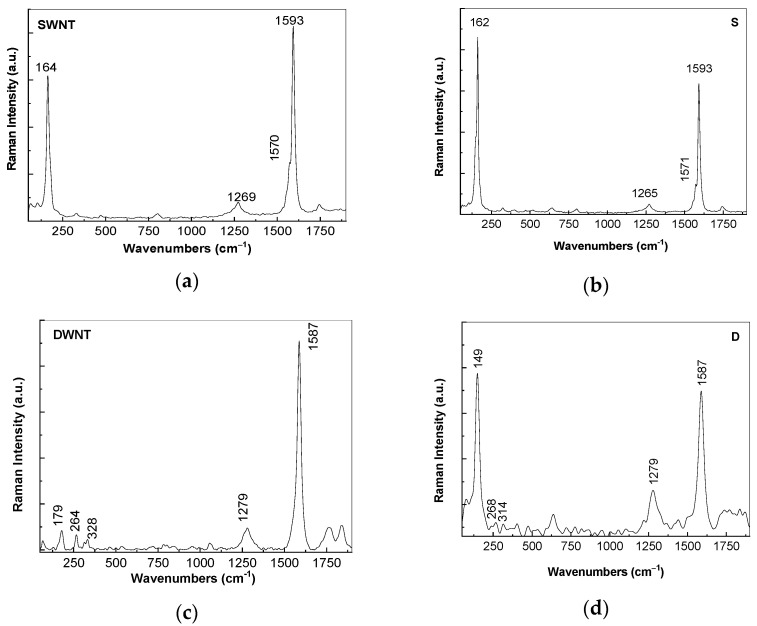

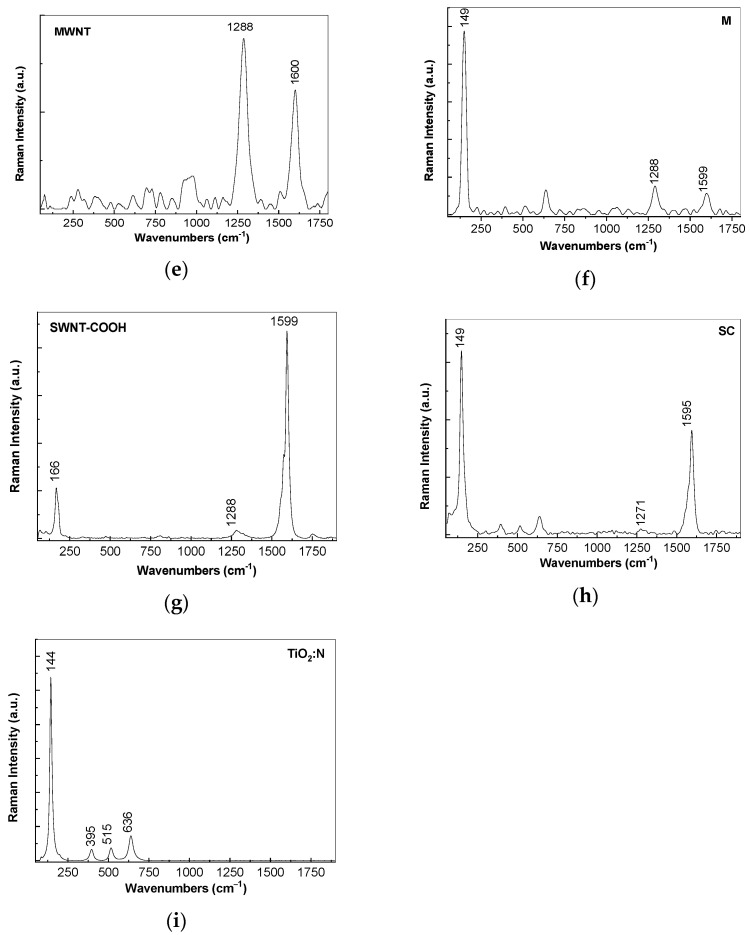
Raman spectra of the samples of SWNTs (**a**), TiO_2_:N/SWNTs (S2 sample) (**b**), DWNTs (**c**), TiO_2_:N/DWNTs (D sample) (**d**), MWNTs (**e**), TiO_2_:N/MWNTs (M sample) (**f**), SWNTs-COOH (**g**), TiO_2_:N/SWNTs-COOH (SC sample) (**h**), and TiO_2_:N (**i**) recorded at the excitation wavelength of 1064 nm.

**Figure 5:**
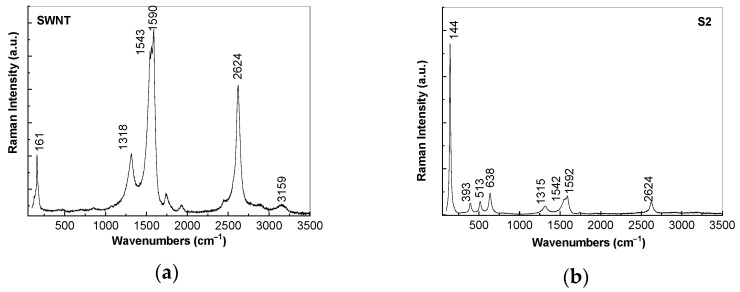

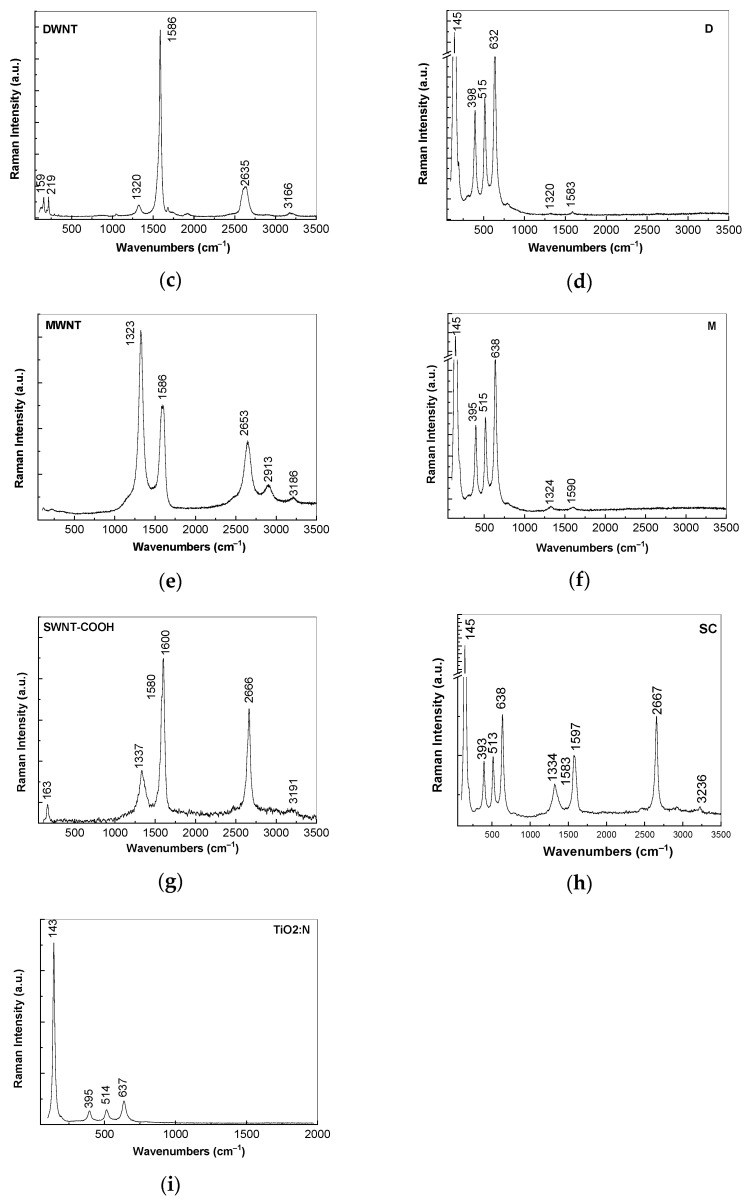
Raman spectra of the samples of SWNTs (**a**), TiO_2_:N/SWNTs (S2 sample) (**b**), DWNTs (**c**), TiO_2_:N/DWNTs (D sample; (**d**)), MWNTs (**e**), TiO_2_:N/MWNTs (M sample; (**f**)), SWNTs-COOH (**g**), TiO_2_:N/SWNTs-COOH (SC sample; (**h**)), and TiO_2_:N (**i**), recorded at the excitation wavelength of 633 nm.

In order to confirm if a chemical adsorption took place between TiO_2_:N nanoparticles and carbon nanotubes of the types SWNTs, DWNTs, MWNTs, and SWNTs-COOH, Figure 4 and Figure 5 show the Raman spectra of SWNTs, DWNTs, MWNTs, and SWNTs-COOH and the S2, D, M, and SC samples, recorded at the excitation wavelengths of 1064 and 633 nm, resonant for semiconductor and metallic tubes, respectively. In the case of a physical adsorption of carbon nanotubes on the surface of TiO_2_:N nanoparticles, in the Raman spectra of the TiO_2_:N/carbon nanotube composites, a sum effect of the Raman lines of the two constituents should be observed. According to the changes reported below, the changes in position and intensity of the Raman lines of the two constituents, i.e., TiO_2_:N and carbon nanotubes, indicate a chemical adsorption of carbon nanotubes on the surface of TiO_2_:N nanoparticles. According to Figure 4, the main Raman lines of the carbon nanotubes are localized in the following spectral ranges:

(i) 100–350 cm^−1^, which are assigned to the radial breathing mode [25]; the maxima of Raman lines are peaked in the cases of SWNTs, DWNTs, and SWNTs-COOH at 164 cm^−1^, 179 cm^−1^–264 cm^−1^–328 cm^−1^, and 166 cm^−1^, respectively; these Raman lines were often used to calculate the diameter of carbon nanotubes [25]. In this context, using (a) the equation ν (cm^−1^) = 248/d (nm) [25], the diameters of the SWNTs and SWNTs-COOH samples are calculated to be 1.51 nm and 1.49 nm, respectively; and (b) using the equations Ω_RBM_ = 218.5/d_inner_ + 15.9 cm^−1^ and d _outer_ = d_inner_ + 0.66 (nm) [26], the inner and outer diameters of DWNTs are equal to (b_1_) 0.7 nm and 1.36 nm, respectively, in the case of Raman line at 328 nm; (b_2_) 0.94 nm and 1.6 nm, respectively, in the case of Raman line at 264 cm^−1^; and (b_3_) 1.38 nm and 2.04 nm, respectively, in the case of Raman line at 179 cm^−1^.
(ii) 1000–1600 cm^−1^, where are observed two Raman bands, labeled as the D and TM band, these being assigned to the disorder state or the defects in the graphitic lattice of carbon nanotubes and the tangential mode, respectively [25]. In the case of SWNTs, DWNTs, MWNTs, and SWNTs-COOH, the maximum of the D band is peaked at 1269, 1279, 1288, and 1281 cm^−1^, respectively, while the maximum of the TM band is situated at 1593, 1587, 1600, and 1593 cm^−1^, respectively.

Raman lines of the TiO_2_:N nanoparticles peaked at 144, 395, 515, and 636 cm^−1^, being assigned to the E_g(1)_, B_1g(1)_, A_1g_, and E_g(3)_ vibrational modes [27,28,29]. The following changes are remarked in the following cases: (i) S2 sample, a down-shift of the Raman lines from 164 cm^−1^ and 1269 cm^−1^ (Figure 4a) to 162 cm^−1^ and 1265 cm^−1^ (Figure 4b), respectively, simultaneously with the change of the ratio between the intensities of the RBM and TM Raman lines (I_RBM_/I_TM_) from 0.75 (Figure 4a) to 1.35 (Figure 4b); (ii) D sample, an up-shift of the Raman lines from 264 cm^−1^ (Figure 4c) and 144 cm^−1^ (Figure 4i) to 268 cm^−1^ and 149 cm^−1^ (Figure 4d), respectively, accompanied by a down-shift of the Raman lines from 328 cm^−1^ (Figure 4c) to 314 cm^−1^ (Figure 4d) and a change of the ratio between the intensities of the D and TM bands (I_D_/I_TM_) from ~0.1 (Figure 4c) to 0.37 (Figure 4d); (iii) M sample, a change of the ratio between the intensities of the D and TM bands of MWNTs (I_D_/I_TM_) from 1.44 (Figure 4e) to 1.3 (Figure 4f), the E_g_ vibrational mode of the TiO_2_:N nanoparticles being peaked at 149 cm^−1^; and (iv) SC sample, the change of the ratio between the intensities of the D and TM bands of SWNTs-COOH (I_D_/I_TM_) from 0.03 (Figure 4g) to 0.06 (Figure 4h) and the down-shift of the D band from 1288 cm^−1^ (Figure 4g) to 1271 cm^−1^ (Figure 4h); the Raman line at 149 cm^−1^ belongs to the TiO_2_:N nanoparticles. These variations indicate in the case of (i) the D and M samples, a higher disorder state in the graphitic lattice of semiconducting tubes highlighted by the increase in the intensity of the D band, as a consequence of adsorption of carbon nanotubes onto the TiO_2_:N nanoparticles surface; and (ii) the S2 and SC samples, a compression induced to semiconducting carbon nanotubes [30] in the presence of the TiO_2_:N nanoparticles evidenced by the down-shift of the D band.

The Raman spectra (Figure 5) highlights in the case of (i) SWNTs, a line with the maximum at 161 cm^−1^ belonging to the RBM vibrational mode (characteristic of the diameter of 1.54 nm), a D band at 1318 cm^−1^ assigned to the disorder state or defects induced to the graphitic lattice of carbon nanotubes, a TM band with the maximum at 1590 cm^−1^ having an asymmetric profile of Breit–Wigner-Fano (BWF) type at ca. 1543 cm^−1^, as well as two lines at 2624 and 3159 cm^−1^, belonging to the vibrational mode of second order of the D and TM bands (2D and 2TM) [25]. The origin of BWF was reported to have the origin in the electron–phonon interaction [31]; (ii) DWNTs, two Raman lines belonging to the RBM vibrational modes of the inner/external diameter of nanotubes equal to 1.53/2.19 nm and 1.07/1.73 nm, which are peaked at 159 cm^−1^ and 219 cm^−1^, respectively, the D and TM bands peaked at 1320 cm^−1^ and 1586 cm^−1^, respectively, and last but not least the 2D and 2TM bands at 2635 cm^−1^ and 3166 cm^−1^, respectively; (iii) MWNTs, the D, TM, 2D, D + M, and 2TM bands are peaked at 1323 cm^−1^, 1586 cm^−1^, 2653 cm^−1^, 2913 cm^−1^, and 3186 cm^−1^, respectively; and (iv) SWNTs-COOH, the RBM, D, TM, 2D, and 2TM bands are localized at 163 cm^−1^ (tube diameter being equal to 1.52 nm), 1337 cm^−1^, 1580–1600 cm^−1^, 2666 cm^−1^, and 3191 cm^−1^, respectively. Figure 5i highlights for TiO_2_:N nanoparticles, four Raman lines peaked at 143 cm^−1^, 395 cm^−1^, 514 cm^−1^, and 637 cm^−1^. As seen in Figure 5, the main changes remarked in the case of the S2, D, M, and SC samples are (i) the shift of the E_g(1)_ vibrational mode of TiO_2_:N from 143 cm^−1^ to 145 cm^−1^ with increases in the wall number of carbon nanotubes; (ii) the ratio between the intensities of the D and TM bands (I_D_/I_TM_) is modified in the case of (a) the SWNTs and S2 samples from 0.35 to 0.47; (b) the DWNTs and D samples from 1.64 to 1.05; (c) the MWNTs and M samples from 0.07 to 0.71; and (d) the SWNTs-COOH and SC samples from 0.4 to 0.62; and (iii) the variation of full width at half maximum (FWHM) of the E_g(1)_ vibrational mode in the case of the TiO_2_:N, S2, D, M, and SC samples from 14.91 cm^−1^ to 16.25 cm^−1^, 15.5 cm^−1^, 15.25 cm^−1^, and 24.94 cm^−1^. Returning to Figure 4, a change in FWHM of the E_g(1)_ vibrational mode is obtained by the deconvolution of the Raman spectra of the TiO_2_:N, D, M, and SC samples from 15.48 cm^−1^ to 31.92 cm^−1^, 28.29 cm^−1^, and 24.85 cm^−1^. Such a variation of FWHM of the E_g(1)_ vibrational mode suggests a change in the local structure of the TiO_2_:N lattice, around the Ti^4+^ ion, as a consequence of an incorporation of carbon nanotubes in the TiO_2_:N matrix. These variations indicate clearly a chemical adsorption of carbon nanotubes onto the TiO_2_:N nanoparticle surface.

### 2.2. Photocatalytic Properties of the TiO_2_:N/CNTs Samples

Photocatalytic activity of the TiO_2_:N and TiO_2_:N/CNTs samples was investigated through RhB photodegradation. Photocatalytic reactions on TiO_2_:N surfaces were described using the Langmuir–Hinshelwood model. All the degradation experiments of RhB performed in this study followed pseudo-first-order kinetics [32]. Effects of SWNT amounts on photocatalytic degradation efficiency were investigated using the TiO_2_:N/SWNT samples with CNT concentrations between 0.25 wt.% and 5 wt.% (samples S1–S3) to optimize the best catalytic system for the degradation of RhB. Degradation efficiency was calculated from the absorption UV–VIS spectra after the exposure to UV light for 300 min, using the following equation:

(1) $$D_{\mathrm{eff}}=\left(\frac{A_{init}-A_{t}}{A_{init}}\right)\times 100$$

where $A_{t}$ and $A_{init}$ are the absorbance values of the analyzed sample corresponding to irradiation times *t* = 10–300 min and *t* = 0 min, respectively.

The degradation rate constant of RhB was calculated using the following expression:

(2) $$\ln\left(\frac{A_{init}}{A_{t}}\right)=k\times t$$

where $A_{t}$ and $A_{init}$ are the absorbance values of the analyzed sample at *t* = 10–300 min and *t* = 0 min, respectively, and *k* represents the degradation rate constant of the reaction at t moment of time.

#### 2.2.1. The Dependence of the RhB Photodegradation on CNT Concentrations in TiO_2_:N/SWNT Samples

**Figure 6:**
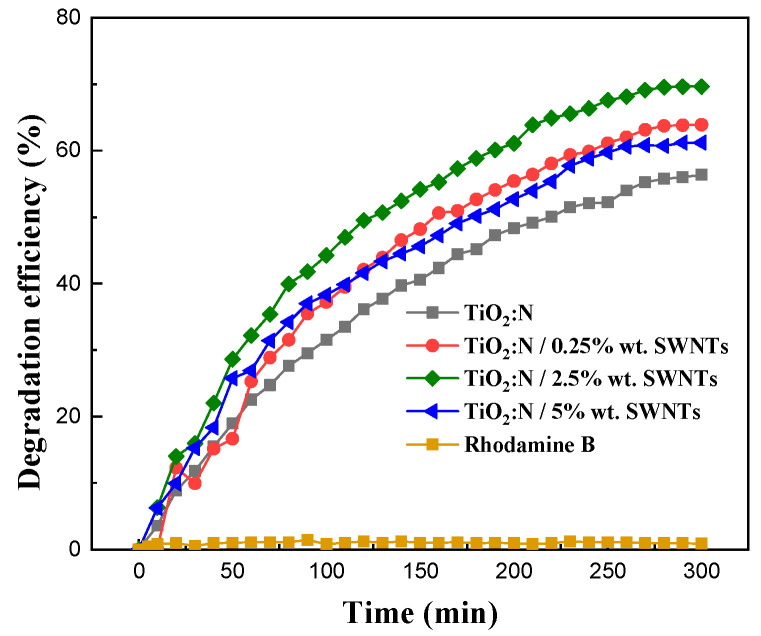
Degradation efficiency of the T and S1–S3 samples dispersed in RhB aqueous solutions after subsequently exposure to UV light.

Figure 6 illustrates the degradation efficiency of RhB for the T and S1–S3 samples as a function of UV light irradiation time, calculated using Equation (1). The obtained results indicate that the best efficiency was depicted in the case of sample S2 when the RhB was approx. 70% decomposed after the exposure to UV light, and a time of 300 min. The presence of SWNTs in the catalyst solution increased the photocatalytic activity, as observed in plots shown in Figure 6. A better analysis of Figure 6 indicates that the degradation efficiency of RhB increased with the SWNT concentration up to 2.5 wt.% CNTs (red and green curves), but at higher SWNTs concentration (5 wt.%) the degradation efficiency decreased (blue curve). As observed in Figure 6, the addition of SWNTs improved the TiO_2_:N photocatalytic activity, the values of the rate constant *k* certifying again this hypothesis.

**Figure 7:**
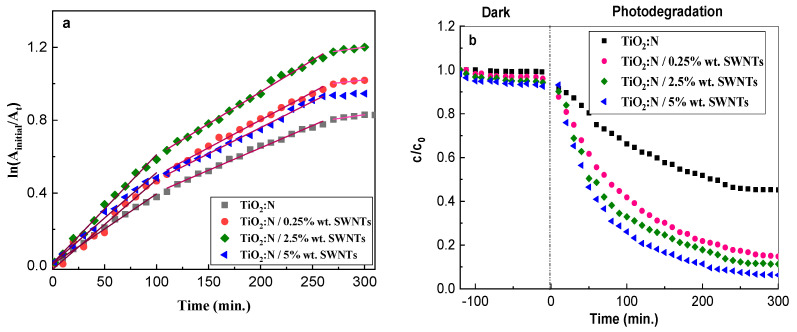
Reaction kinetics of the RhB aqueous solutions in the presence of the T and S1–S3 samples and UV light (**a**). Photocatalytic degradation of RhB solution in the presence of the T and S1–S3 samples (**b**).

Figure 6 and Figure 7a,b clearly indicate that the RhB photodegradation is dependent on the concentration of carbon nanotubes in the TiO_2_:N/SWNTs mixtures. The experimental data plotted in Figure 7a revealed three linear regions; hence they do not fit a simple first-order kinetic model.

The reaction rate constant values for each sample ($k_{1}$, $k_{2}$, and $k_{3}$) and the corresponding linear regression coefficients ($R_{1}^{2}$, $R_{2}^{2}$, and $R_{3}^{2}$) are presented in Table 2.

**Scheme 1:**
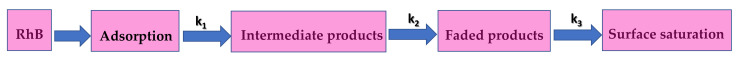
The stages of the RhB photocatalytic degradation.

From Table 2, it can be observed that the rate constants increased with SWNT concentration up to the *k* value of 0.0055 min^−1^ in the case of 2.5 wt.% SWNTs, which was due to a higher loading of carbon nanotubes onto the TiO_2_:N nanoparticle surface. By increasing SWNT concentration to 5 wt.% in the TiO_2_:N/SWNT composite, the rate constant *k* decreased, the value of this constant being superior to that reported in the case of the TiO_2_:N nanoparticles. The linear regression coefficients had values ranging from 0.7059 to 0.9958, which indicate that RhB photodegradation took place, in agreement with the first-order kinetics. The fastest photodegradation was obtained in the case of Sample S2. The presence of the three linear regions in Figure 7a can be explained if we consider that the RhB photocatalytic degradation was made in three steps according to the following stages shown in Scheme 1 [33,34]:

In the first step, RhB was absorbed on the TiO_2_:N or TiO_2_/SWNT sample surface, and after the exposure to UV light the organic dye was degraded to intermediate products, with rate constant *k*_1_. The intermediate products were transformed in faded products having the rate constant *k*_2_. In the final step the values of *k*_3_ rate constant indicated the saturation of the sample surfaces. The increase of RhB degradation efficiency when SWNTs were added to TiO_2_:N can be attributed to faster electron–hole recombination with increases of the SWNT concentration in the TiO_2_:N/SWNT mixture [21]. A higher SWNT concentration can block the irradiation and obstruct charge carrier generation; in addition, SWNTs can form aggregates due to van der Waals interactions between individual tubes and act as a screening agent for TiO_2_:N, blocking its exposure to UV light. [21]. This will reduce the production of reactive oxygen species, which are essential for RhB degradation. If the SWNT concentration is too high (5 wt.%), TiO_2_:N particles are insufficiently covered, and therefore photons are absorbed and scattered by SWNTs, inducing the decrease of the sample photoactivity.

**Figure 8:**
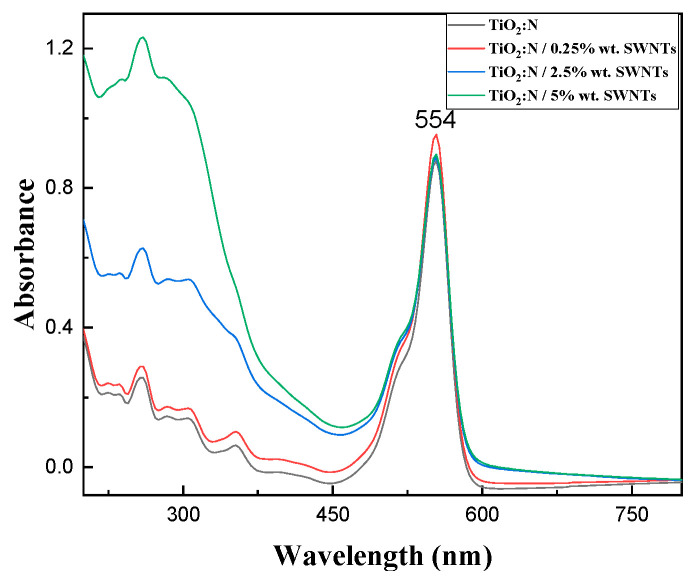
UV–VIS absorption spectra of the T and S1–S3 samples dispersed in an RhB aqueous solution in dark conditions, i.e., before to exposure to UV light.

Figure 8 illustrates the UV–VIS absorption spectra of the T and S1–S3 samples dispersed in RhB aqueous solution, before exposure to UV light in which approximately similar values of absorbance were obtained. This fact can be explained based on the hypothesis that a similar amount of photons are absorbed regardless of SWNT concentration in the TiO_2_:N/SWNTs samples, which verifies the conclusion illustrated in the above discussion that SWNTs improve the RhB photocatalytic degradation. The absorption band situated at 554 nm corresponds to π–π* transitions of RhB [35].

#### 2.2.2. Influence of the Carbon Nanotubes Type on the RhB Photodegradation

**Figure 9:**
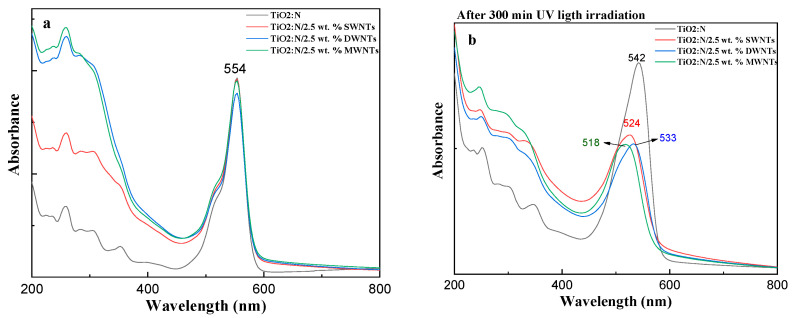
The UV–VIS spectra of the RhB aqueous solutions containing the T, S2, D, and M samples, before (**a**) and after (**b**) to exposure to UV light time of 300 min.

Figure 9a highlights that regardless of the carbon nanotubes type, i.e., SWNTs, DWNTs, or MWNTs, the presence of carbon nanotubes (with the concentration of 5 wt.%) into TiO_2_:N/CNTs samples dispersed in RhB aqueous solutions does not induce large variations in the UV–VIS spectra of the samples recorded prior to exposure to UV light.

**Figure 10:**
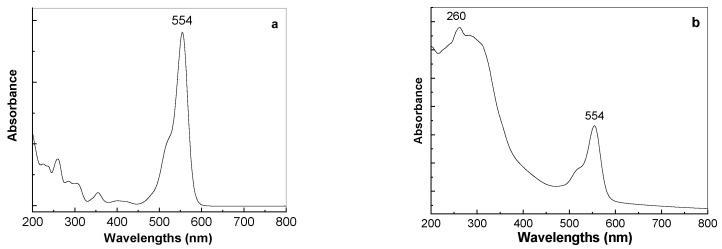
The UV–VIS spectra of the RhB aqueous solutions alone (**a**) and containing DWNTs (**b**).

The UV–VIS spectra of the T, S2, D, and M samples were dominated by the band peaked at 554 nm, having a shoulder at approx. 515 nm, characteristic of RhB. After the subsequent exposure to UV light up to 300 min of the RhB aqueous solutions containing the T, S2, D, and M samples, we observed the following: (i) a down-shift of the RhB absorption band from 554 nm to 542 nm, 524 nm, 533 nm, and 518 nm, respectively, in the case of aqueous solutions of RhB containing (a) TiO_2_:N and its mixtures with (b) 2.5 wt.% SWNTs, (c) 2.5 wt.% DWNTs, and (d) 2.5 wt.% MWNTs, respectively; (ii) the disappearance of the shoulder situated at 515 nm simultaneously with the widening of the main absorbance band of RhB; and (iii) a decrease in the absorbance of band situated at 554 nm from approx. 0.96 to (a) 0.42 in the case of the T sample, (b) 0.26 for sample S2, and (c) 0.25 in the case of the D and M samples. These modifications and the invariance of the RhB absorbance of the band peaked at 554 nm before exposure to UV light of the TiO_2_:N/SWNT, TiO_2_:N/DWNT, and TiO_2_:N/MWNT samples support the hypothesis that carbon nanotubes enhance the photocatalytic activity of TiO_2_:N. RhB can transfer to the TiO_2_:N/CNT catalyst’s surface, and it can be absorbed by π–π conjugation between the RhB and carbon hexagonal ring of CNTs, increasing the absorptivity of RhB on CNTs compared to that of RhB on bare TiO_2_:N. In order to explain the high absorbance of the band with the maximum at 260 nm in the case of samples of TiO_2_:N mixed with carbon nanotubes, in Figure 10 are shown the UV–VIS spectra of RhB and of the sample containing 5 mg DWNTs in 10 mL RhB 100 μM. Figure 10a,b demonstrates that the origin of the band localized in the spectral range 200–400 nm is in the adsorption of RhB onto the DWNs surface, when the π–π* bonds are established between the benzene rings of RhB and graphitic structure of carbon nanotubes.

**Figure 11:**
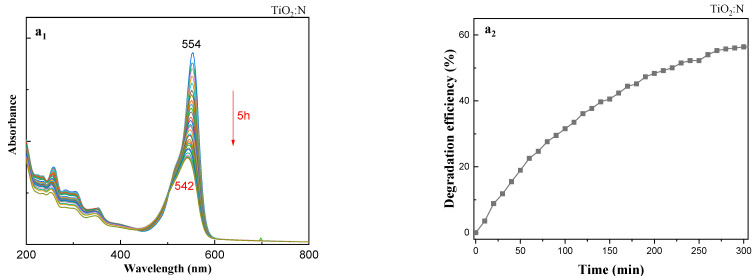

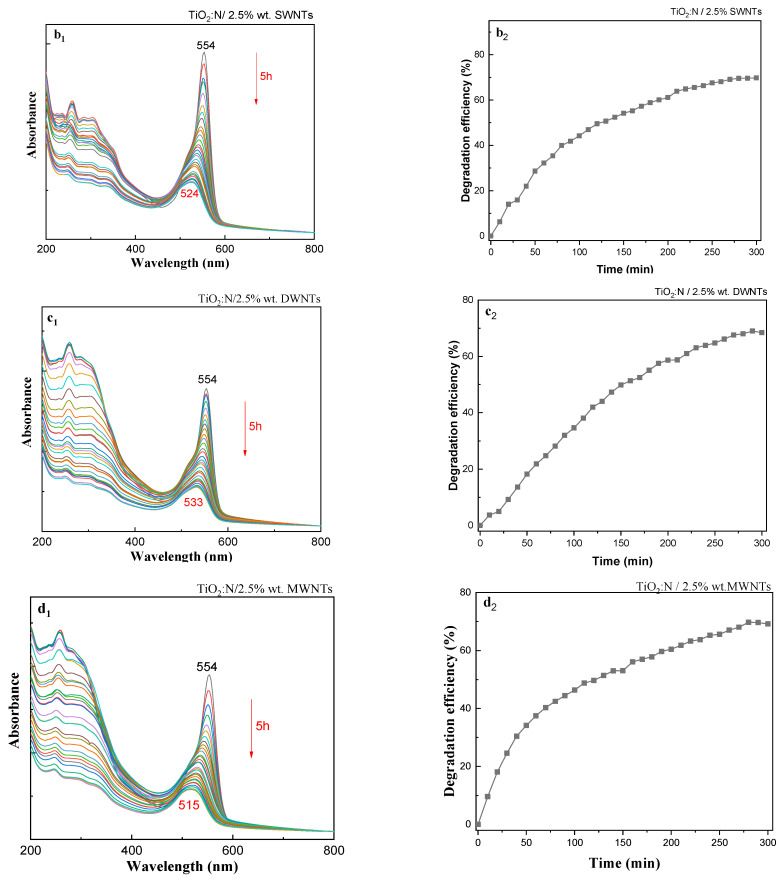
UV–VIS spectra of the RhB aqueous solutions containing T, S2, D, and M samples recorded by subsequence exposure to UV light time of 300 min (**a_1_**,**b_1_**,**c_1_**,**d_1_**); the dependence of the RhB degradation efficiency with the time of exposure to UV light in the presence of T, S2, D, and M samples (**a_2_**,**b_2_**,**c_2_**,**d_2_**).

The detailed evolution of RhB degradation in aqueous solutions containing the T, S2, D, and M samples after the subsequent exposure to UV light and the degradation efficiency of RhB depending on the irradiation time is illustrated in Figure 11. To further the analysis of RhB degradation efficiency in aqueous solutions containing the T, S2, D, and M samples, respectively, using Equation (1), the plots in Figure 11(a_2_–d_2_) were obtained.

RhB photodegradation in aqueous solutions containing the S2, D, and M samples could be analyzed based on the values obtained for reaction constants *k*_1_, *k*_2_, and *k*_3_, respectively. From Table 3, one can observe various rate constant values when the colloidal suspensions contained different types of carbon nanotubes. Taking into account the RhB photocatalytic degradation steps invoked in Scheme 1, the three-step model could also be applied in the case of the S2, D, and M samples. As described before *k*_1_ indicates the rate constant value assigned to the formation of intermediate products; therefore, from Table 3, we can observe that the highest value was found in the case of the TiO_2_:N/MWNT sample, indicating that a faster process in the first stage takes place. This result may be due to the presence of MWNTs, which provide a higher specific interfacial area for photon absorption so that inner and outer walls can both absorb oxygen in solutions [22]. *k*_2_ corresponds to the solution discoloration, and the corresponding values illustrated a higher discoloration capacity in the case of samples based on TiO_2_:N/DWNT and TiO_2_:N/MWNT composites. The third step of the RhB photodegradation mechanism was deduced by the *k*_3_ rate values, indicating the saturation of final products. Additionally, *k*_3_ rate values presented in Table 3 indicate that the saturation process took place in similar conditions for all the samples. Table 3 highlights the rate constants superior to that reported for the TiO_2_:N nanoparticles (T sample in Table 2), the *k*_1_ value ranging from 0.00364 min^−1^ (T sample) to 0.0055 min^−1^ (S2 sample), 0.0046 min^−1^ (D sample), and 0.0084 min^−1^ (M sample), the result which indicates the highest loading of carbon nanotubes on the surface of TiO_2_:N nanoparticles occurred in the case of the M sample.

**Figure 12:**
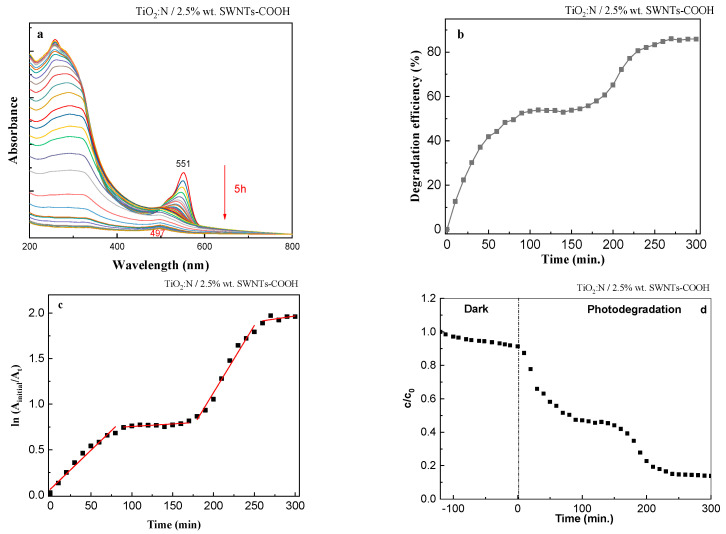
UV–VIS absorption spectra of the RhB aqueous solution containing the SC sample, recorded by the successively exposure to UV light, time of 300 min (5 h) (**a**), the degradation efficiency as a function of the UV irradiation time (**b**), and the reaction kinetics (**c**). Photocatalytic degradation of RhB solution in the presence of the SC sample and UV light (**d**).

As shown in the following, noticeable results were obtained in the case of the TiO_2_:N/SWNTs-COOH samples. The exposure to UV light for 300 min of the RhB aqueous solution containing TiO_2_:N and 2.5 wt.% SWNTs-COOH implied significant modification in the UV–VIS spectra illustrated in Figure 12a: (i) a down-shift of the band of RhB from 551 nm (t = 0 min) to 497 nm (t = 300 min); (ii) the disappearance of the band shoulder situated at 515 nm; and (iii) the gradual decrease of the absorbance band situated at 551 nm from 0.69 to approx. 0.09. According to Figure 12b, the RhB photodegradation has a sinusoidal behavior and after 50 min of UV irradiation, the degradation efficiency reached rapidly 50% and continued up to 85% after 300 min of exposure to UV light.

Figure 12c illustrates a different profile from the results presented in the case of RhB aqueous solutions containing the T and S1–S3 samples (Figure 7), indicating that the RhB photodegradation process took place in four stages. After fitting the plot from Figure 12c, the rate constant values *k*_1_, *k*_2_, *k*_3_ and *k*_4_ were equal to 0.008, 0.0005, 0.0147, and 0.0013 min^−1^, respectively. In the case of the SC, the value of the rate constant *k*_1_ equal to 0.008 min^−1^ was higher than that reported in the case of sample S2, which was equal to 0.0055 min^−1^; this fact indicates that the presence of carboxyl functional groups onto SWNTs surface facilitated the loading of nanotubes on the surface of TiO_2_:N nanoparticles. This four-stage process might be explained based on the fact that after 170 min of UV irradiation there were unreacted species in the sample solution. Continuing with the irradiation, new chemical bonds between TiO_2_:N and SWNTs-COOH were formed, and therefore as a consequence, the *k*_3_ rate constant value was higher than *k*_1_, indicating better electron transfer between TiO_2_:N and SWNTs-COOH. After 5 h UV irradiation, the sample solution was colorless. CNTs tend to aggregate in aqueous solutions; therefore, the CNT functionalization could increase the hydrophilicity and the homogeneous dispensability in the TiO_2_:N matrix. The presence of the COOH groups on the SWNTs groups enhanced the chemisorbed oxygen species on the CNTs surface and promoted the oxidation capacity of the TiO_2_:N catalyst. According to [36], the catalytic performance depends on the activity of surface-OH groups, and additionally it has a strong dependence on the concentration of surface OH radicals. The COOH groups from functionalized SWNTs broaden the specific surface area and improve the adsorption capacity of TiO_2_:N/SWNTs catalyst [37].

**Figure 13:**
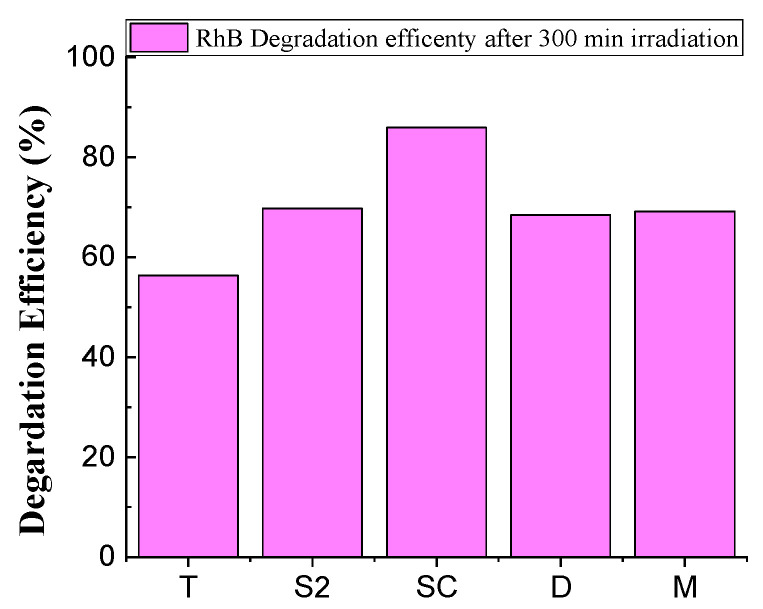
Photodegradation efficiency of the RhB aqueous solutions in the presence of T, S2, SC, D, and M samples after 300 min of UV light irradiation.

Based on the results of this present study regarding the photodegradation efficiency of RhB in the presence of samples containing TiO_2_:N and different types of CNTs, the diagram from Figure 13 was created. Figure 13 indicates that after 300 min of UV light irradiation, the highest degradation efficiency was obtained in the case of samples containing TiO_2_:N and 2.5 wt.% SWNTs-COOH, which ensured RhB photodegradation with more than 80% in the aqueous solution. The improved photocatalytic activity of the TiO_2_:N/2.5 wt.% SWNTs-COOH sample can be explained due to the homogeneous and dense dispersion of TiO_2_:N nanoparticles on the CNT surface. The strong contact between TiO_2_:N and CNTs induce dense heterojunctions through Ti-O-C structures [23]. The functional groups COOH in SWNTs increase the active chemisorbed oxygen amount; therefore, both the capability of oxygen delivery and the oxygen mobility on the lattice surface was also improved. The presence of SWNTs-COOH in the TiO_2_:N/SWNTs-COOH composite induced a synergy effect of TiO_2_:N, mainly attributed to the strong binding by the functional groups of carboxylic acid.

#### 2.2.3. Mechanism of the RhB Photocatalytic Degradation Enhanced in the Presence of CNTs

**Scheme 2:**
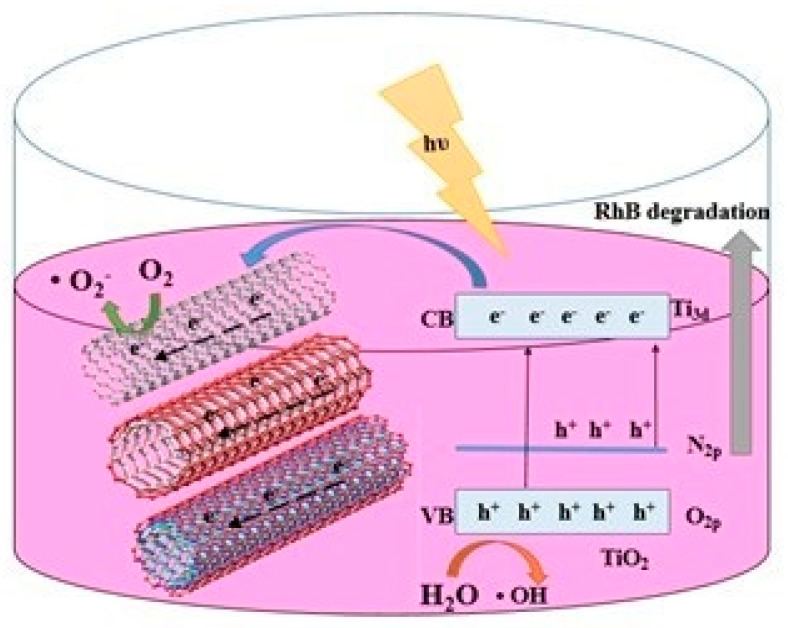
The mechanism of RhB photocatalytic degradation in the presence of the TiO_2_:N/CNT samples.

RhB photodegradation in the presence of TiO_2_:N sample has more than 50% efficiency due to the doped semiconductor. Nitrogen doping of TiO_2_ allows shallow states within the semiconductor band-gap; therefore, new energetic levels are introduced between the valence and conduction bands, resulting in a narrowing bandgap than the undoped TiO_2_ [38]. Doping TiO_2_ with non-metal elements can generate new oxygen vacancies and increase the density of the catalyst [23]. The UV radiation excites the electrons in both the mid-gap energy level and the valence band of TiO_2_:N [39]. The enhancement of the photocatalytic activity of TiO_2_:N/CNTs composites can be explained using different mechanisms: (i) the mechanism proposed by Hoffmann et al. [40] states that the incoming energy photon excites an electron from the valence band to the conduction band of TiO_2_, and then the photogenerated electrons are transferred to CNTs with the remaining holes participating in redox reactions; (ii) the mechanism proposed by Wang et al. [24] recalls that CNTs act as sensitizers and transfer the electrons to TiO_2_. The photogenerated electron is transferred to the TiO_2_ conduction band and allows the formation of superoxide radicals; furthermore, the CNTs are positively charged and have the ability to remove one electron from the TiO_2_ valence band, leaving a hole. The resulting positively charged TiO_2_ can react with adsorbed water and form hydroxyl radicals; and (iii) the mechanism in which CNTs can act as impurity forming Ti-O-C bonds similar to carbon-doped titania [41]. The mechanism of RhB photocatalytic degradation in aqueous solutions containing TiO_2_:N/CNT samples is illustrated in Scheme 2.

CNTs can enhance the photocatalytic efficiency of TiO_2_:N due to the electrons’ adsorption and transportation. The mid-gap energy level induced in TiO_2_ after the nitrogen doping is situated close to the valence band of TiO_2_. Under the exposure to UV light of the samples containing TiO_2_:N, electron (*e^−^*) and hole (*h^+^*) pairs are generated. Further *e^−^* are excited from the valence band and from the mid-gap energy level to the conduction band of TiO_2_:N, creating holes. According to Nila et al. [38], the highest energy level of the TiO_2_:N valence band is −6.05 eV, with a TiO_2_:N band gap of 2.3 eV. When TiO_2_:N particles are in strong contact with *CNTs*, the energy levels of TiO_2_:N can superpose to the HOMO-LUMO levels of *CNTs*, inducing the transfer of electrons from TiO_2_:N surface to the LUMO level of *CNTs*. From the LUMO level of *CNTs*, electrons can suffer successive transitions until the minimum *LUMO* energy level from which they are collected by the maximum energy level of *HOMO*, hindering the recombination of *e^−^*–*h^+^* pairs. *e*^−^ from the *CNTs* surface react with water and oxygen leading to hydroxyl radical which will oxidize the RhB. The photodegradation of *RhB* can be described as follows:

(3) $$LUMO\left(e^{-}\right)\to HOMO\left(e^{-}\right)$$

(4) $$TiO_{2}:N+CNTs\;\overset{UV\;irradiation}{\Rightarrow }e_{CB}^{-}+h_{VB}^{+}+h_{MG}^{+}\to CNTs\;\left(e^{-}\right)+\left(h^{+}\right)$$

(5) $$TiO_{2}:N\left(h_{VB}^{+}+h_{MG}^{+}\right)+OH_{ads}^{-}\to \cdot OH+TiO_{2}:N$$

(6) $$TiO_{2}:N\left(e_{CB}^{+}\right)+O_{2\;ads}\to \cdot O_{2}^{-}+TiO_{2}:N\overset{H_{2}O}{\to }\cdot OH+TiO_{2}:N$$

(7) $$CNTs+\left(e^{-}\right)+O_{2\;ads}\to \cdot O_{2}^{-}+CNTs\overset{H_{2}O}{\to }\;\cdot OH+CNTs$$

(8) $$RhB_{ads}+\;\cdot OH\to \mathrm{Degradation}$$

**Figure 14:**
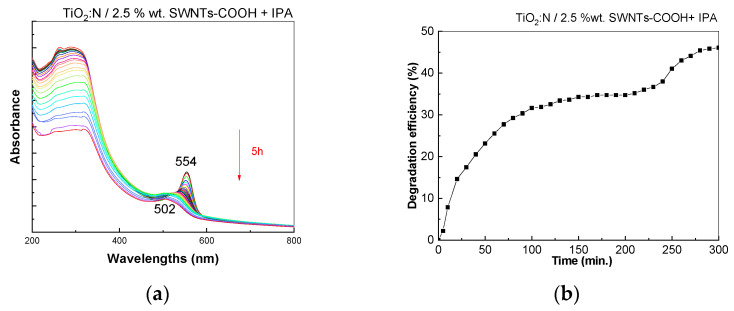
UV–VIS absorption spectra of the RhB aqueous solution containing the SC sample and IPA, recorded by the successively exposure to UV light, time of 300 min (5 h) (**a**), and the degradation efficiency as a function of the UV irradiation time (**b**).

To highlight that the reactive species, i.e., hydroxyl radicals and O_2_, play a significant role in the RhB photodegradation process when the TiO_2_:N/carbon nanotube samples are used as catalysts, isopropyl alcohol (IPA) was used as quencher. Figure 14a shows the UV–VIS spectra of the RhB aqueous solution containing the SC sample and IPA as scavenger. Prior to exposure of the sample to UV light, the UV–VIS spectrum was characterized by a band with a maximum at 554 nm and another localized in the spectral range 200–400 nm. The exposure to UV light of the RhB aqueous solution containing the SC sample and IPA, in a time of 300 min, induced a gradual decrease in the absorbance of the band belonging to RhB, simultaneously with a shift from 554 nm to 502 nm. Figure 14b highlights similar behavior with that shown in Figure 12b, the degradation efficiency after 300 min of exposure to UV light being equal to 46%, a value lower than that reported in the absence of IPA. This result indicates that the hydroxyl radicals play a significant role in the photodegradation process of the RhB aqueous solution containing the SC sample.

Taking into account the above results, we can conclude that (i) the samples prepared in this work show high photocatalytic activity when a small concentration of carbon nanotubes (2.5 wt.%) is added to the TiO_2_:N matrix; and (ii) a high degradation efficiency of the RhB solution is obtained in the case of the TiO_2_:N/SWNTs-COOH sample.

## 3. Materials and Methods

In this paper, TiO_2_:N with a nitrogen concentration of 1.5% wt. was purchased from NANO-X GmbH, having purity of 99%. RhB, IPA, and carbon nanotubes (SWNTs, SWNTs-COOH, DWNTs, and MWNTs) were purchased from Sigma-Aldrich (St. Louis, MO, USA).

The TiO_2_:N/SWNTs samples with different SWNT concentrations (0.25, 0.5, 1, 2.5, and 5 wt.%) were prepared by direct mixing of the two constituents. In the case of the TiO_2_:N/SWNTs-COOH, TiO_2_:N/DWNT, and TiO_2_:N/MWNT samples, the CNT concentration was 2.5 wt.%. The samples were labeled as described in Table 1. The colloidal dispersions were prepared by adding each sample to an aqueous solution of RhB 100 μM followed by magnetic stirring for 20 min at 25 °C. Each suspension was stirred in dark conditions to reach the adsorption–desorption equilibrium of RhB on the catalyst surface. The colloidal dispersions were subjected to subsequent UV light irradiation for 300 min with a halogen lamp of 300 W.

UV–VIS spectra of the solutions were recorded with a Perkin Elmer Lambda 950 UV–VIS–NIR spectrophotometer (PerkinElmer, Inc., Waltham, MA, USA).

Raman spectra of SWNTs, DWNTs, MWNTs, SWNTs-COOH, TiO_2_:N, and their composites S_1_–S_3,_ D, M, and SC were recorded using (i) an FT Raman spectrophotometer, MultiRam model, from Bruker, endowed with a YAG:Nd laser, the excitation wavelength being of 1064 nm (Billerica, MA, USA); and (ii) a Raman spectrophotometer, T64000 model, from Horiba Jobin Yvon (Palaiseau, France), endowed with a solid laser from Cobolt, the excitation wavelength being of 633 nm (Kassel, Germany).

SEM images of the S_2_, D, M, and SC samples were recorded with a Zeiss Gemini 500 scanning electron microscope (Zeiss, Oberkochen, Germany).

TEM (transmission electron microscopy) investigations of the S_2_, D, M, and SC samples were performed with a JEOL ARM 200F microscope (JEOL (Europe) SAS).

## 4. Conclusions

This paper reports new results obtained by UV–VIS absorption spectroscopy concerning RhB photodegradation in the presence of TiO_2_:N and TiO_2_:N/CNT composites, under UV light. In this work, characterization of the TiO_2_:N/CNTs composites was performed by SEM, TEM, and Raman scattering. Our results led to the following conclusions: (i) values of E_g_ in the case of the samples TiO_2_:N, S2, D, M, and SC, having a carbon nanotubes concentration of 2.5 wt.%, are equal to 3.17 eV, 3.04 eV, 3.06 eV, 3.15 eV, and 3.12 eV, respectively; (ii) the SEM analysis highlighted in the case of the S2, D, M, and SC samples, both TiO_2_:N platelets and one-dimensional structures corresponding to the bundles of SWNTs, DWNTs, MWNTs, and SWNTs-COOH; (iii) according to TEM analysis, mean size of TiO_2_:N nanoparticles in the S2, D, M, and SC samples was assessed to be equal to 28.4 nm, 28.81 nm, 26.11 nm, and 26.43 nm, respectively; (iv) the Raman studies evidenced changes of FWHM of the E_g(1)_ vibrational mode assigned to the Raman line at ~144 cm^−1^ in the case of the TiO_2_:N, S2, D, M, and SC samples from 14.91 cm^−1^ to 16.25 cm^−1^, 15.5 cm^−1^, 15.25 cm^−1^, and 24.94 cm^−1^, as a consequence of incorporation of carbon nanotubes in the TiO_2_:N matrix, a fact which induces an increase in the disorder state of the graphitic lattice of carbon nanotubes; (v) the reaction kinetics of RhB aqueous solutions in the presence of TiO_2_:N/SWNTs samples having various CNT concentration followed a complex first order kinetic model made in three stages. The addition of SWNTs in the TiO_2_:N matrix increases the photocatalytic activity in the case of samples containing 0.25 and 2.5 wt.% SWNTs due to the CNTs’ capacity to hinder the electron–hole recombination. At higher SWNT concentrations (i.e., 5 wt.%), the RhB degradation efficiency decreases because SWNTs can form aggregates due to van der Waals interactions between individual tubes, acting as a screening agent for TiO_2_:N under UV irradiation; (vi) the exposure to UV light up to 300 min of the RhB aqueous solutions in the presence of the TiO_2_:N/CNT (SWNTs, DWNTs, and MWNTs) samples induces a down-shift of the RhB absorption band from 554 nm to 524 nm, 533 nm, and 518 nm, respectively, indicating that RhB is adsorbed to the TiO_2_:N surface; (vii) after 300 min of exposure to UV light, the TiO_2_:N/SWNTs-COOH catalyst induced the down-shift of the absorbance band of RhB from 551 nm to 497 nm simultaneously with the gradual decrease of its absorbance, indicating an efficient RhB photodegradation. The presence of SWNTs-COOH in the TiO_2_:N/SWNTs-COOH samples led to RhB degradation with 85% and induced a synergy effect of TiO_2_:N attributed to the strong binding by the functional –COOH group; (viii) the highest RhB photodegradation efficiency after 300 min of exposure to UV light was observed in the case of samples containing TiO_2_:N/SWNT composites (approx. 80%), followed by samples containing TiO_2_:N and CNTs of the type of SWNTs, DWNTs, and MWNTs (approx. 70%). Therefore, CNTs can enhance the photocatalytic efficiency of TiO_2_:N due to the electron adsorption and transportation, leading to a hindering of electron–hole recombination. In comparison with other works which report a degradation efficiency of methylene blue, methylene orange, and RhB dyes of ~38%, ~33%, and ~70%, respectively, in the presence of TiO_2_/MWNTs, obtained by hydrolysis and hydrothermal methods [17,42], in this work we have demonstrated that (i) by using the method of mixing the two constituents, which is cheaper and faster, values of a RhB photodegradation efficiency in the presence of the catalysts TiO_2_:N/SWNTs, TiO_2_:N/DWNTs, and TiO_2_:N/MWNTs of 70%, 68%, and 69%, respectively, are reported when the concentration of carbon nanotubes is only 2.5 wt.% in the mass of mixtures; and (ii) the best catalyst for the efficient photodegradation of the RhB solution is the TiO_2_:N/SWNTs-COOH sample; the photodegradation efficiency of the RhB solution in the presence of TiO_2_:N/SWNTs-COOH sample is of 85%, a value higher than that reported in the case of photodegradation of methylene blue in the presence of the TiO_2_:N/MWNTs-COOH catalyst, which was a maximum of 60% [23].

## Figures and Tables

**Table 1:** Short names of the samples used in the study.

Samples	Labeled Name in the Study
TiO_2_:N	Sample T
TiO_2_:N/0.25 wt.% SWNTs	Sample S1
TiO_2_:N/2.5 wt.% SWNTs	Sample S2
TiO_2_:N/5 wt.% SWNTs	Sample S3
TiO_2_:N/2.5 wt.% SWNTs-COOH	Sample SC
TiO_2_:N/2.5 wt.% DWNTs	Sample D
TiO_2_:N/2.5 wt.% MWNTs	Sample M

**Table 2:** Reaction kinetics constants of the RhB aqueous solutions in the presence of the T and S1–S3 samples.

Sample Name	$k_{1}$ (min^−1^)	$R_{1}^{2}$	$k_{2}$ (min^−1^)	$R_{2}^{2}$	$k_{3}$ (min^−1^)	$R_{3}^{2}$
T	0.00364	0.9935	0.0023	0.9913	0.0008	0.9864
S1	0.0047	0.9832	0.0028	0.9958	0.0006	0.7878
S2	0.0055	0.9895	0.0034	0.9906	0.0008	0.9451
S3	0.0044	0.9795	0.0029	0.9943	0.0004	0.7059

**Table 3:** Reaction kinetic constants of the S2, D, and M samples dispersed in the RhB aqueous solutions.

Sample Name	*k*_1_ (min^−1^)	$R_{1}^{2}$	*k*_2_ (min^−1^)	$R_{2}^{2}$	*k*_3_ (min^−1^)	$R_{3}^{2}$
S2	0.0055	0.9895	0.0034	0.9906	0.0008	0.9451
D	0.0046	0.9958	0.0364	0.9951	0.0011	0.5745
M	0.0084	0.9911	0.0273	0.9968	0.0009	0.8635

## Data Availability

The data presented in this study are available on request from the corresponding authors.
